# Coevolution with bacteria drives the evolution of aerobic fermentation in *Lachancea kluyveri*

**DOI:** 10.1371/journal.pone.0173318

**Published:** 2017-03-10

**Authors:** Nerve Zhou, Krishna B. S. Swamy, Jun-Yi Leu, Michael J. McDonald, Silvia Galafassi, Concetta Compagno, Jure Piškur

**Affiliations:** 1 Department of Biology, Lund University, Lund, Sweden; 2 Institute of Molecular Biology, Academia Sinica, Taipei, Taiwan; 3 School of Biological Sciences, Monash University, Melbourne, Australia; 4 Department of Food, Environmental and Nutritional Sciences, University of Milan, Milan, Italy; University of Strasbourg, FRANCE

## Abstract

The Crabtree positive yeasts, such as *Saccharomyces cerevisiae*, prefer fermentation to respiration, even under fully aerobic conditions. The selective pressures that drove the evolution of this trait remain controversial because of the low ATP yield of fermentation compared to respiration. Here we propagate experimental populations of the weak-Crabtree yeast *Lachancea kluyveri*, in competitive co-culture with bacteria. We find that *L*. *kluyveri* adapts by producing quantities of ethanol lethal to bacteria and evolves several of the defining characteristics of Crabtree positive yeasts. We use precise quantitative analysis to show that the rate advantage of fermentation over aerobic respiration is insufficient to provide an overall growth advantage. Thus, the rapid consumption of glucose and the utilization of ethanol are essential for the success of the aerobic fermentation strategy. These results corroborate that selection derived from competition with bacteria could have provided the impetus for the evolution of the Crabtree positive trait.

## Introduction

While glycolysis is fundamental to nearly all organisms, there are multiple metabolic pathways for the utilization of pyruvate, the product of glycolysis. One option is fermentation, which does not require oxygen and converts pyruvate into CO_2_ and ethanol, yielding two molecules of ATP. An alternative fate for pyruvate is the TCA Cycle which generates at least six times more ATP per unit of pyruvate than fermentation, but requires oxygen. It is perhaps not surprising that most cells prefer aerobic respiration to fermentation in the presence of oxygen. However, there are several notable exceptions. The first are populations of cancer cells that overcome the typical repressors of metabolism in what is known as the Warburg effect [1, 2]. Another is the Crabtree positive yeasts [3] such as *S*. *cerevisiae*, which prefer fermentation to respiration in oxygen and sugar rich environments [4–6]. The selective forces that led to the evolution of aerobic fermentation in the Crabtree positive yeasts remain an open question.

One potential explanation derives from the product of fermentation. The make-accumulate-consume (MAC) hypothesis emphasizes that ethanol is lethal to many microbes in concentrations tolerable to Crabtree positive yeasts [5–7]. The rapid conversion of sugar to ethanol has the potential to not only destroy or weaken possible competitors, but also to provide a carbon source that can be further utilized via oxidative respiration. The MAC hypothesis is supported by the functional divergence of a duplicate pair of genes, *ADH1* and *ADH2*, that arose from the whole genome duplication in the common ancestor of Crabtree positive yeasts [8, 9]. *ADH1* encodes an enzyme for the production of ethanol, while its duplicate *ADH2* is unique to Crabtree positive yeasts and encodes an enzyme specialized for the conversion of ethanol to acetyl CoA for subsequent utilization in the TCA cycle [10].

An alternative explanation for the evolution of the Crabtree positive phenotype posits a fundamental rate/yield tradeoff between fermentation and respiration [11]. Although fermentation produces less ATP per molecule of glucose than respiration, its higher rate may allow for more ATP to be produced per unit of time. Even though aerobic fermentation is inefficient, it satisfies the evolutionary imperative to convert nutrients into biomass as fast as possible. The Rate Yield Tradeoff (RYT) hypothesis is attractive as it potentially explains the evolution of the Crabtree positive phenotype without making assumptions about the utility of ethanol, and is supported by several theoretical analyses [11, 12]. Currently, the evidence in support of the MAC and RYT hypotheses has been theoretical or circumstantial, with direct experimental data addressing differences in these two hypotheses difficult to obtain.

Experimental populations of microbes provide a means to directly test evolutionary hypotheses, including the effect of interspecies competition on the evolution of species interactions [13, 14]. Here we exploit a weak Crabtree positive yeast, *Lachancea kluyveri*, to test the conditions that could have led to the evolution of the Crabtree positive phenotype. *L*. *kluyveri* generally favors respiration over fermentation. However, certain conditions can lead to temporary fermentation in the presence of oxygen [15, 16]. We designed an evolution experiment to select on two aspects of fermentation. First, we propagated three replicate lines of *L*. *kluyveri* in a constant state of exponential growth in a high sugar environment, thus selecting for rate over yield, hereafter referred to as the RYT treatment. Second, we propagated three replicate lines of *L*. *kluyveri* in the presence of bacteria to select for the potential bactericidal effect of ethanol produced during fermentation (MAC treatment).

## Results and discussion

### MAC lines evolve elevated ethanol yields more rapidly than RYT lines

**Fig 1:**
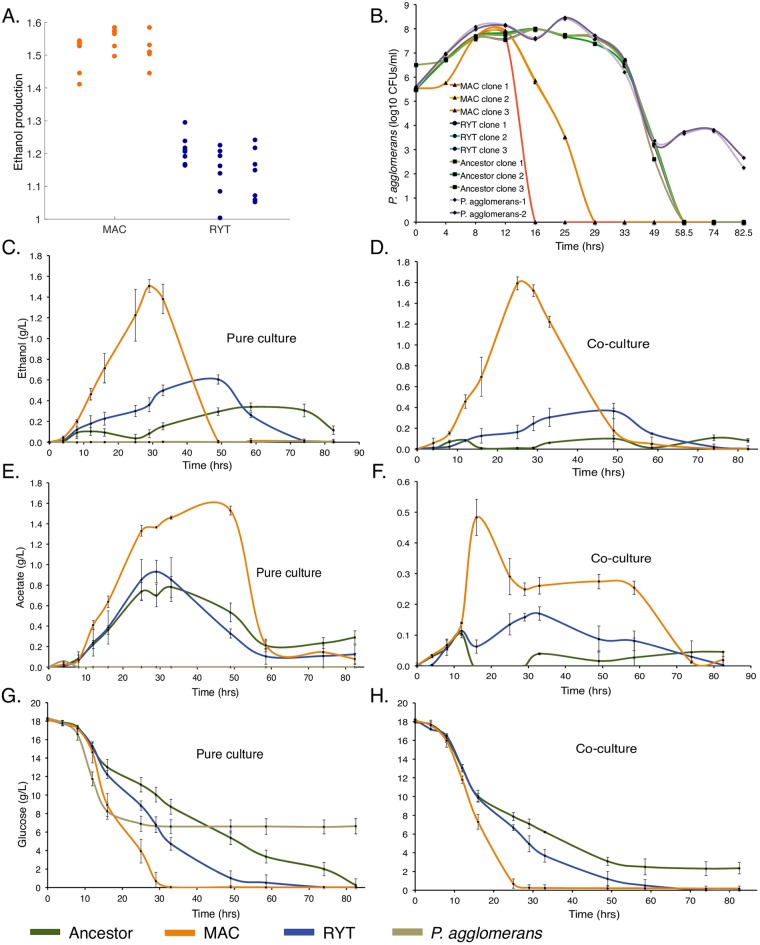
MAC and RYT treatments evolved different metabolite production and consumption profiles. (A) Ratio (fold change) of ethanol yields of clones selected from MAC and RYT with respect to the ancestor is presented here. A fold change more than 1 indicates ethanol yield of ancestor is lower than MAC or RYT strain being compared with. The relative ethanol yield of MAC is significantly greater than RYT and ancestor strains (Mann-Whitney U, *p* < 10^−4^). The fermentative yeast *S*.*cerevisiae* CEN.PK113-7D (0.38g/L) [37] has 1.83 fold higher ethanol yield than the *L*. *kluyveri* ancestor (0.207g/L). (B) Time course of the numbers of *P*. *agglomerans* bacteria during co-culture with MAC (Triangles), RYT (Bullets) and ancestral strains (Squares), the time course of duplicate clones of *P*. *agglomerans* (Diamonds) in absence of yeasts as control (MAC vs RYT and ancestor Mann-Whitney U, *p* < 0.01). The decrease in bacteria can be attributed to reduction in carbon source. Ethanol (C, D) and acetate (E, F) production in MAC pure and co-cultures was significantly greater than RYT and ancestor strains (Mann-Whitney U, *p* < 0.01) and the peak of ethanol production in MAC strains coincides with the time point at which *P*. *agglomerans* was decimated. Glucose (G, H) was consumed by MAC at half the time that of RYT and ancestor strains in pure and co-cultures (Mann-Whitney U, *p* < 0.01). Error bars are one standard deviation and ancestor (green), RYT (blue), MAC (orange) and bacteria (*P*. *agglomerans*) (olive).

MAC treated strains were obtained by propagating *L*. *kluyveri* in YPD in competition with 5 bacterial species of increasing ethanol tolerance, replaced sequentially after every 160 generations. RYT treated strains were obtained using the same dilution regime, but without bacterial competition. After ~720 generations of evolution we evaluated the ethanol production of all experimental lines. We found that MAC populations evolved significantly higher ethanol yields than RYT populations (Mann-Whitney U, *p* < 0.0001), while both MAC and RYT strains produced more ethanol than the ancestor (Mann-Whitney U, *p* < 0.05) (Fig 1A). The ethanol production in MAC strains remained significantly higher than RYT strains till 960 generations, the end of the evolution experiment (S1A Fig). We selected clones from each of the three MAC and three RYT populations and tested the persistence of the bacteria *Pantoea agglomerans* in co-culture with MAC, RYT and ancestral populations. *P*. *agglomerans* was used for all the phenotypic and metabolic assays due to its relative ease to culture as well as its growth rate, which was similar to that of the ancestral yeast. We found that while the ancestor and the RYT clones took between 56–60 hrs to eliminate *P*. *agglomerans* as bacterial competitors, the MAC populations took between 16-29hrs (Fig 1B). The viability of more ethanol tolerant bacteria (S1D–S1F Fig) was also significantly reduced only when co-cultured with MAC clones. Viability of *L*. *kluyveri* populations was not affected (S2 Fig). Although, both MAC and RYT strains have an elevated ethanol yield, bactericidal ability is evident only in higher ethanol producing MAC strains. This suggests that bacterial competition could have provided impetus for evolution of aerobic fermentation. All further experiments were carried out using these clones.

### MAC strains show higher peak ethanol levels and rapid consumption of glucose

In order to determine the cause of the apparent bactericidal activity of MAC strains, we tracked the consumption of glucose and the production of ethanol and acetate over 80 hrs in both pure and co-cultures. MAC strains attained higher peak ethanol concentrations (~5 fold) (Fig 1C and 1D) and acetate (~7 fold) (Fig 1E and 1F) than RYT and ancestral strains. The production of ethanol requires high rates of glucose use. Correspondingly, we found that MAC strains exhausted glucose almost twice as quickly as the ancestor, while the RYT strains also consumed their glucose more quickly than the ancestor (Fig 1G and 1H). As a confirmation of the validity of our measurements, we calculated correlation coefficients for the concentrations of metabolites over time. We found glucose concentrations over the first 33hrs in the MAC and RYT strains to be anti-correlated with ethanol and acetate concentrations (R^2^ > 0.94, *p* < 1×10^−5^)(Fig 1C, 1E, 1G and S3 Fig). This is to be expected during the first phase of glucose consumption since glucose is converted to ethanol or acetate. Cell death was not correlated with ethanol concentrations in both RYT strains (R^2^ = 0.19, *p* = 0.11) and the ancestor (R^2^ = 0.10, *p* = 0.46), but highly correlated in MAC strains tested (R^2^ = 0.96, *p* < 1.03×10^−5^) implicating higher ethanol concentrations in the extinction of competing bacteria (Fig 1B and 1D).

### Bacterial death is not due to carbon source restriction

It is possible that *L*. *kluyveri* restricts bacterial growth by limiting access to glucose. We found that at 16 hrs, when bacterial populations began to decline, residual glucose was at 40% of inoculation levels (7.3 ± 0.8 g/L) while at the time of bacterial extinction, glucose levels remained at 4% (0.71 ± 0.5 g/L) (Fig 1H), suggesting that the unavailability of glucose was not the likely cause of bacterial extinction. Analysis of the acetate profiles in pure cultures of MAC strains shows the accumulation and maintenance of over 1.5 g/L of acetate between 20 to 50 hrs (Fig 1E). In mixed cultures acetate levels decline significantly as glucose approaches exhaustion. Interestingly, the decline in acetate levels stops at 30 hrs and plateaus, coinciding with bacterial extinction (Fig 1F). This suggests that bacteria use acetate as well as glucose in mixed cultures, and were still actively utilizing acetate until their extinction. We measured the growth rates of our bacterial strains at a range of acetate concentrations, and found that *P*. *agglomerans* could grow on 1g/L acetate as a sole carbon source (S1H Fig). In all co-cultures, bacterial viability was measured by counting colony-forming units on agar plates. While lack of a carbon source would slow growth, it should not cause death. If bacterial cells had depressed growth levels due to carbon source restriction, plating on agar should have allowed such cells to recover. Together, these data corroborate that carbon source restrictions are not the cause of bacterial cell death in MAC clones co-cultures.

### Aerobic fermentation does not confer an advantage during consumption of glucose in strictly aerobic conditions

The RYT and MAC hypotheses posit different advantages to fermentation. The RYT hypothesis suggests that the advantage of aerobic fermentation over respiration comes from superior ATP yields per unit time during the consumption of glucose. The MAC hypothesis predicts that the benefit of aerobic fermentation derive from the utilization of ethanol. The key point of difference is that if the advantage of fermentation can be measured during glucose consumption, and before ethanol is utilized, then the RYT hypothesis provides a sufficient explanation of the Crabtree positive phenotype [11].

**Fig 2:**
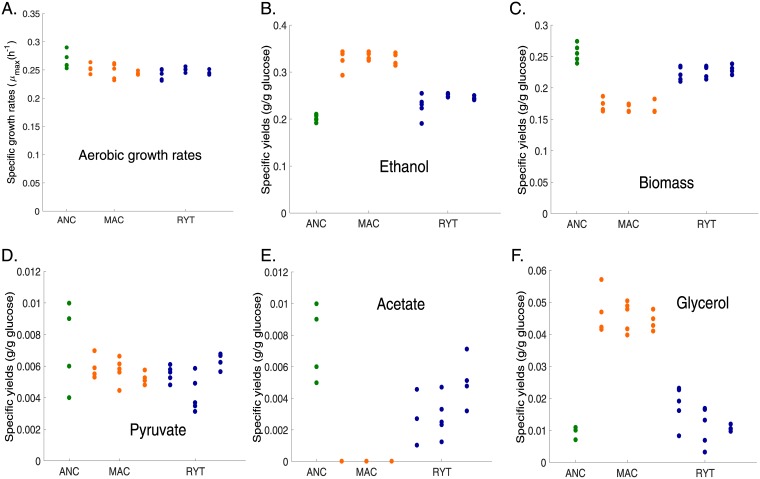
MAC and RYT differences are specific to aerobic conditions. Fermentation parameters of the ancestor (green), RYT (blue) and MAC (orange) strains are shown for strictly aerobic condition. Growth rates calculated for ancestral, RYT and MAC strains in strictly aerobic conditions (Mann-Whitney U, *p* < 0.05). (A). Specific product yields of the ancestor, MAC and RYT for ethanol (B), biomass (C), pyruvate (D), acetate (E), and glycerol (F) in strictly aerobic conditions. All the observed differences are significant (Mann-Whitney U, *p* < 0.05).

To test the RYT and MAC hypotheses we compared the growth rates and biomass yields of the ancestor, MAC and RYT strains in controlled fermenters. This allowed the measurement of strains in strictly aerobic, anaerobic or the experimental growth conditions (Fig 2, S4 and S5 Figs). Production of biomass has been shown to be strictly dependent on ATP yields [17], so we took our growth rate estimates as a proxy for ATP yield. We found that during the utilization of glucose, and under strictly aerobic conditions MAC strains had significantly reduced growth rates and biomass production compared to the ancestor (Mann-Whitney U, *p* < 0.003 and *p* < 0.009 respectively) (Fig 2A and 2C). Conversely, in experimental growth conditions where strains could utilize the ethanol that they had produced by fermentation (S1B and S1G Fig), the MAC strains had significantly higher growth rate than the ancestor (Mann-Whitney U, *p* < 0.01), while RYT strains did not. MAC strains also had higher growth rates than the ancestor (Mann-Whitney U, p < 0.01) in strictly anaerobic conditions and RYT strains (Mann-Whitney U, p < 0.05), where it had to solely depend on fermentation (S4 Fig). This suggested that that MAC strains could utilize ethanol more efficiently than RYT strains. Thus, in contradiction to the prediction of the RYT hypothesis, the advantage garnered by either RYT or MAC strains depended upon being able to utilize the ethanol that they had produced.

**Fig 3:**
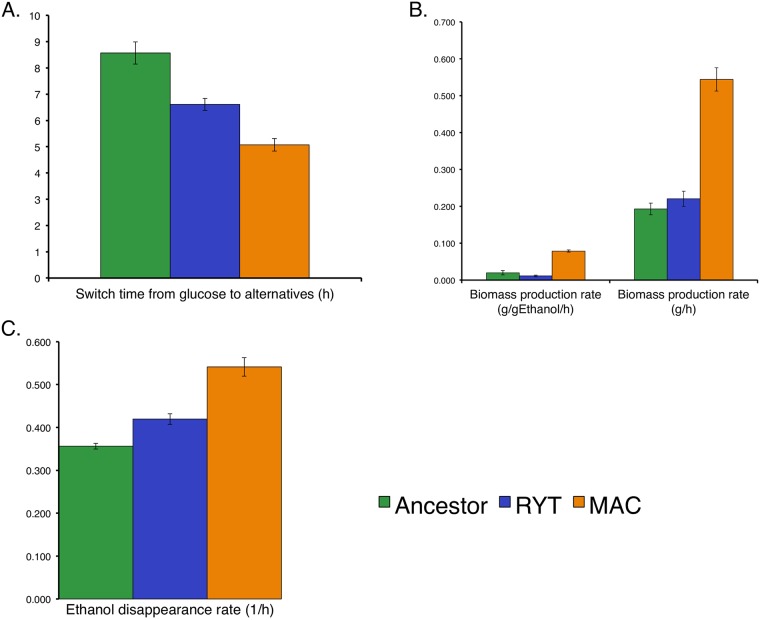
Post-diauxic condition comparative physiology of yeasts. (A) MAC (orange) swiftly switched from glucose to alternative carbon source in a significantly shorter time as compared to ancestor (green) and RYT (blue) strains (t-test, *p* < 0.01). (B) MAC demonstrated higher biomass production (t-test, *p* < 0.01) and (C) consumed ethanol faster than all strains (t-test, *p* < 0.01). MAC is associated with a fitter phenotype in post-diauxic phase based on these parameters.

We measured the speed of switching to ethanol from glucose and the efficiency of converting ethanol into biomass (Fig 3). We found that both MAC and RYT strains could switch to ethanol hours faster than the progenitor strain and that the MAC strain could get more biomass per gram of ethanol. These results suggest that the advantage to fermentation is speed, but in a different form to that posited by the RYT hypothesis. Instead, Crabtree positive yeast must not only produce ethanol, but also be quick to switch from fermentative glucose metabolism to respiratory ethanol utilization, and be efficient in converting ethanol into ATP/biomass.

### Metabolite profiles in strict fermentation conditions resemble Crabtree positive yeast

In order to look at further differences between treatments we interrogated the production and consumption of metabolites in MAC and RYT treatment strains in controlled fermentation conditions. We found that all differences were amplified in strictly aerobic cultures, while in the anaerobic conditions, in which the ancestral *L*. *kluyveri* is obligated to ferment glucose, RYT and MAC were nearly indistinguishable from the progenitor strain in most of the measures (Fig 2, S4B Fig). Although we had earlier detected high levels of acetate production in the experimental growth conditions, under strict aerobic conditions MAC strains had almost no detectable levels of acetate and elevated levels of glycerol (Fig 2, S6 Fig). These results suggest that the differences that we measure between the MAC and RYT treatments are mainly due to changes in aerobic and not anaerobic metabolism.

**Fig 4:**
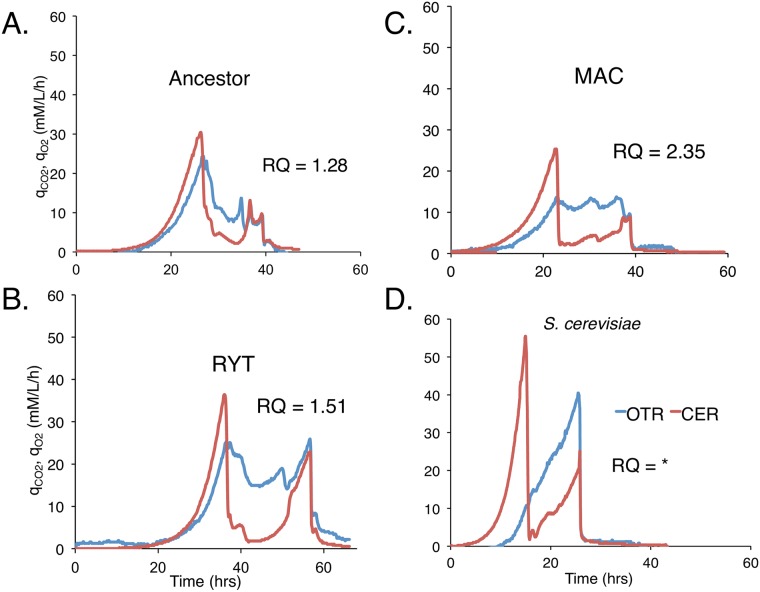
CO_2_ Evolution Rate (CER) and O_2_ Transfer Rate (OTR) profiles for aerobic batch fermentations of the (A) ancestor, (B) RYT, (C) MAC and (D) *S*. *cerevisiae*. * RQ value for *S*. *cerevisiae* strains differ from strain to strain but our laboratory strain (CEN.PK 113-7D) has an RQ value of 4.48 ± 0.72. Respiratory activity was calculated from data compiled from bioreactors fitted with exhaust gas analyzers and mass transfer equation was used to calculate OTR and CER (mM/l/h). Oxygen uptake before the diauxic shift decreased over time and in general MAC seems to have evolved to be less dependent on O_2_. The first drop in CER at 25.5 hrs (ancestor), 35.3 hrs (RYT), 22 hrs (MAC) and 14.7 hrs (*S*. *cerevisiae*) coincides with glucose exhaustion. The second drop in CER indicates depletion of alternative carbon sources. The plateau of OTR in MAC profile represents the maximum transfer rate of the system, implying reduction in oxygen demand during the growth phase.

A key measure of an aerobic or anaerobic metabolism is the ratio of CO_2_ production to O_2_ consumption, or respiratory quotient (RQ). An RQ value of 1 indicates a fully aerobic metabolism, typical of Crabtree negative yeast. Crabtree positive yeast such as *S*. *cerevisiae* have a RQ of 2, or higher [18]. We calculated RQ values for the ancestor and the RYT strains and found them to be in agreement with previous measurements for *L*. *kluyveri* [18]. All three MAC strains were found to have significantly higher RQ values, in the range of measurements for *S*. *cerevisiae* (Fig 4 and S5 Fig). Next, we tracked CO_2_ production and O_2_ consumption over time. *L*. *kluyveri* is characterized by a close coupling of oxygen consumption and CO_2_ production, as expected for an aerobe. RYT clones, and to a greater degree, MAC clones had decoupled CO_2_ production from oxygen consumption, in a marked shift toward the profile of *S*. *cerevisiae* (Fig 4).

### RNASeq shows selection on glycolysis

To reveal the gene expression changes underlying the Crabtree positive adaptations of MAC strains, we carried out RNASeq (in triplicate) on ancestral, MAC and RYT clones taken from three conditions: aerobic, post-diauxic shift and anaerobic. A gene was considered misregulated if the absolute value of fold change in MAC and RYT genes was greater than or equal to 1.5 (S7 Table). Gene ontology analysis was performed on the misregulated gene to get global information about the transcriptomic data (S4 Table). A wide array of functional categories were enriched during the analysis, with generalized relevant categories being carbohydrate transport and metabolism, response to stress, cell cycle and cell wall maintenance, cellular respiration, mitochondrial organization, generation of precursor metabolites and energy.

**Fig 5:**
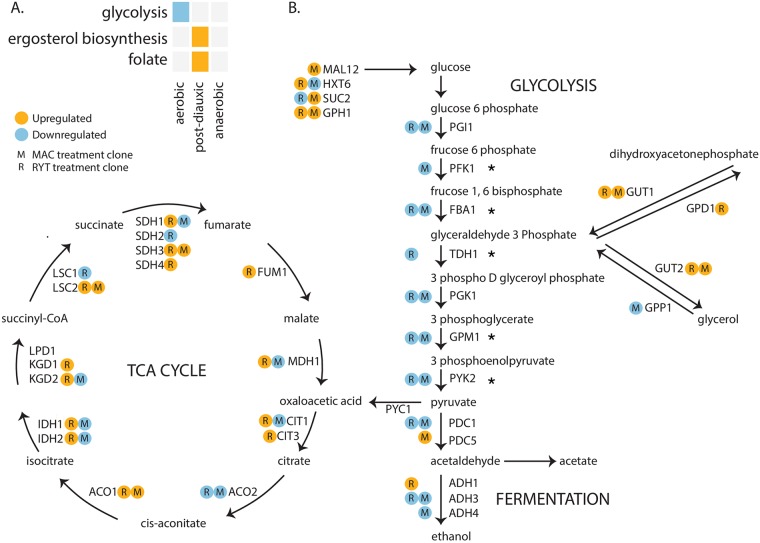
Rapid gene expression evolution in glycolysis, the TCA cycle and fermentation. (A) The pathways with significant directional change of genes. (B) The expression level of genes encoding core enzymes in glucose metabolism, under aerobic growth conditions. Circles to the left or right of an arrow indicate an upregulation (orange circle) or downregulation (blue circle). * denote pathways having multiple enzymes.

With up to 1300 genes being misregulated and a broad range of gene class functions enriched, it is difficult to ascertain meaningful genes responsible for the observed phenotype. Thus, we designed a test for selection based on the directional changes of gene expression across biochemical pathways (Fig 5, S1 Table), considering only those changes that were consistent to both RYT and MAC conditions. Among all metabolic pathways, we found the strongest signal in glycolysis, with significant down regulation of glycolysis in both MAC and RYT lines in aerobic conditions (Fig 5). It seems intuitive that down regulation of glycolytic genes should result in decrease in glycolytic flux. However, five of the eight steps in glycolysis have multiple enzymes, it maybe that changes in the ratios of these enzymes caused by deregulation and altering of the contributions for other glucose metabolism pathways results in increased glycolytic flux. Alternatively, it is possible that the glycolytic pathway is downregulated to maintain homeostasis of glycolytic intermediates, as their accumulation can be toxic for the cell [19]. We find the pyruvate decarboxylase *PDC1* downregulated in both aerobic and diauxic conditions in MAC and RYT strains and *PDC5* upregulated only in MAC strains in all the three conditions. The increase in ethanol yield in MAC strains could thus be attributed to the overexpression of *PDC5*, which could have efficiently reduced pyruvate into acetaldehyde and was in turn converted into ethanol [20].

Whole genome sequencing of one of the ancestor and MAC clones (Ancestor clone 1 and MAC clone 1 in Fig 1B) and extensive searches for SNPs and indels on this data and all the RNASeq data from MAC and RYT strains were performed. An overview of sequencing statistics is provided in S2 Table. While, MAC strains contained 11 nonsynonymous, 2 inframe indels, 6 synonymous and 9 intergenic SNPs in 18 genes, the RYT strains contained 14 non-synonymous and 18 synonymous SNPs in 23 genes (S3 Table). The nonsynonymous variants detected in MAC strains were in the *S*. *cerevisiae* orthologus genes *DOA1*, *VTA1*, *BNI1*, *PIR1*, *PDR12*, *APN1*, *NMD3*, *RPL33A* (S3A Table). Some of these variants were relevant in stress response, DNA damage and cell wall integrity. *DOA1*, a Cdc48 adaptor with ubiquitin binding domain had a nonsense mutation. It is a critical mediator of the turnover of mitochondrial Cdc48 substrates. *doa1Δ* mislocalization and accumulation of mitochondrial substrates [21, 22]. *PIR1* a gene with important roles in cell wall integrity had nonsynonymous mutations. The accelerated increase in ethanol production in MAC strains can be toxic for the cell and is also known to increase DNA damage. We found nonsynonymous mutations in PIR1, a gene important in cell wall integrity pathway [23] and the endonuclease *APN1* involved in repair of DNA damage [24]. Incidentally, Pir1 is also important for translocation of Apn1 into mitochondria to maintain genomic stability [25]. However, no mutations in known regulators of glucose metabolism were found.

For the RYT strains we performed extensive searches for indels and SNPs on RNASeq data from all the three conditions. The variants listed in S3B Table are those found in all the 3 conditions. The nonsynonymous variants in RYT strains were found in 11 genes orthologus to *S*. *cerevisiae* (S3B Table). The relevant variants were in DNA replication stress and DNA damage response (*RAS1*, *RAS2*, *RSR1*) [26, 27] and cell wall integrity pathway (*PTS1*, *ECM33*) [28, 29], ER maintenance and oxidative stress (*SRP102*) [30]. Some of these pathways were similar between MAC and RYT strains, suggesting these pathways are the primary response when fermentative ability of yeast is increased.

Further work is required to track down the genetic causes of the phenotypic and gene expression changes that we found here. Also, the observed transcriptional signatures can be radically different from proteomic expression levels and the observed phenotypes could be due to epigenetic or post-translational modifications. A whole proteome analysis of the evolved strains can probably provide evidence of the causal loci.

Together our results corroborate that the advantage that Crabtree positive yeast get from aerobic fermentation depends upon the utilization of ethanol after diauxic shift, providing evidence against the RYT hypothesis. The large amount of expression changes we found in glycolysis suggests strong selective forces at work in both RYT and MAC lines. However, the presence of bacterial competitors provided stronger selection, driving MAC strains to a phenotypic profile that more closely resembled Crabtree positive yeasts. While encouraging, further work is required to determine and demonstrate the genetic changes that underlie the evolution of the Crabtree positive phenotype.

## Materials and methods

### Strains used in the study

*Saccharomyces (Lachancea) kluyveri* CBS 3082 (NRRL Y-12651 diploid type strain) was used as the ancestral strain for our laboratory evolution experiments. The following bacterial strains were used in the evolution strategy: *Pantoea agglomerans* Eh318 (CUCPB 2140), *Serattia plymuthica* AS9 (CCUG 61396), *Bacillus subtilis* PS216, *Streptomyces venezuelae* (ATCC 10712) and *Lactococcus lactis* (NCDO 2118).

### Determination of ethanol toxicity

Effects of ethanol on bacterial viability used to evolve the ancestral yeast were tested by exposing bacteria to LB media supplemented with 3, 6, 9, 10, 11, or 12% (v/v) ethanol (adjusted to pH 7.4). Samples were withdrawn and bacterial densities were determined by measurement of OD_600 nm_ using a Helios spectrophotometer (Spectronic Unicam, Cambridge, UK). Cell viability after exposure to ethanol was determined by estimating colony-forming units (CFUs). The effects of ethanol on the *L*. *kluyveri* ancestral strain were examined by exposure to ethanol for 3, 5 and 10 days (in YPD media supplemented with 3, 6, 9, 10, 11, or 12% (v/v) ethanol followed by plating on solid YPD (adjusted to a pH of 6.2) to test cell viability (S5 Table).

### Evolution experiment

A single colony of *L*. *kluyveri* was used to inoculate six baffled-bottomed flasks containing 20mL of YPD and incubated at 25°C with shaking Three of these experimental lines, designated RYT, were diluted 1:400 into fresh media every 48 hrs. This dilution regime did not allow cultures to enter stationary phase. The three other experimental lines, designated MAC, followed the same dilution regime as RYT treatment strains, but were interrupted after 4 hrs of incubation by inoculation with bacteria (4 ± 0.05 log_10_ cfu/mL) and at 44 hrs by adding Streptomycin (100 μg/mL) in order to cure the bacteria. We determined that streptomycin did not affect yeast survival (S1I Fig).

After 160 generations (20 transfers), bacteria known to be inhabiting soil and fruits environments were replaced sequentially, in this order *Pantoea agglomerans*, *Serattia plymuthica*, *Bacillus subtilis*, *Streptomyces venezuelae*, and *Lactococcus lactis*; based on the order of ethanol tolerance we had determined (S5 Table). Before each transfer cycle we froze 500μL of cell culture suspension in 25% glycerol at −80°C for further analyses. To ensure no contamination, we sequenced rDNA of the D1/D2 region of *L*. *kluyveri* strains, amplified by universal primers NL1 (5’-GCATATCAATAAGCGGAGGAAAAG-3’) and NL4 (5’- GGTCCGTGTTTCAAGACGG-3’) before proceeding with the next bacterium.

### Culture conditions for metabolite and co-culture analysis

Yeast and bacteria co-cultivation experiments were done aerobically in 25 mL YPD (2% glucose, 0.5% yeast extract and 1% peptone), at a pH of 6.2 in 250 mL baffled-bottom shake flasks at 25°C at 200 r.p.m in an Infors HT Ecotron shaker unit (Infors HT). We co-inoculated 5 ± 0.05 log_10_ cfu/mL yeast and 4 ± 0.05 log_10_ cfu/mL bacteria and then monitored growth and viability for at least 3 days. At predetermined time intervals, aliquots of 0.5 mL were withdrawn to examine metabolites and determine viability. Cell viability of both bacteria and yeast in co-culture was determined by estimating CFUs following plating on selective YPD media containing 100 μg/mL Streptomycin to select against bacteria or 10 μg/mL cycloheximide-supplemented LB media to select against yeasts. All the 5 bacterial species used to evolve yeast strains were then competed with either of the evolved yeasts as well as with the ancestral yeast. *Pantoea agglomerans* was then used to extensively study yeast strains in competition experiments due to its relative ease to culture as well as its growth rate, which was similar to that of the ancestral yeast. Controls with pure yeast or bacteria were included. All experiments were done in triplicate.

### Batch cultivation in fermenters

Unless otherwise specified all aerobic batch cultivations of three isolates from each evolution line were performed in triplicate using Multifors (Infors HT) fermenters with a working volume of 1 L. We used synthetic defined minimal media [31]. Dissolved oxygen (monitored by an InPro 6800S sensor from Mettler Toledo) was maintained above 30% by an inbuilt automation system that varied the stirring speed between 200 and 1200 r.p.m. at 25°C. Airflow was set at 1 L/min. We maintained pH at 5(± 0.5) by automatic addition of 2M KOH and 1M H_2_SO_4_ and monitored with a 405-DPAS-SC-K8S/225-pH sensor (Mettler Toledo). Gas analyzers BC-CO_2_ and BCP-O2 (Blue Sens) were fitted on the exhaust line to determine CO_2_ and O_2_ concentrations in the outflow in the respective order. Seed cultures were grown overnight in 100 mL minimal medium in 500 mL baffled-bottom flasks at 25°C and 200 r.p.m., washed and used to inoculate all batch cultures as previously reported [31].

Anaerobic experiments were performed with the same fermenter units, instead fitted with Norprene tubings (Cole-Parmer) to reduce diffusion of O_2_ and flushed with N_2_ (< 3 ppm O_2_) at a flow rate of 0.1 L N_2_/min at a constant steering speed of 300 r.p.m. Synthetic minimal media was supplemented with 420 mg/L Tween-80, 50 mg/L uracil and 10 mg/L ergosterol. All fermenter experiments were done in triplicates. After fermentation, we sampled from each of the fermenters and streaked the yeasts on a solid YPD plate before picking colonies and subsequently verified them by sequencing of the rDNA D1/D2 region amplified by universal primers reported in the section “Evolution experiment” above.

### Growth kinetics and extracellular metabolite analyses

We monitored cell growth by concurrent measurement of dry weights (DW) and optical density (OD). We used DW to calculate maximum specific rates of metabolites production (for example ethanol, acetate, and CO_2_) and consumption of glucose and oxygen. Glass microfibre GF/A filters (Whatman) were weighed before and after filtering a known amount of sample, washed with distilled water, and then dried in an oven at 70°C for a minimum of 24 h to determine cell dry weights. All growth kinetics studies were done with glucose as the sole carbon source. Cell growth was also monitored in parallel by measuring OD_600nm_.

We sampled the fermenters during exponential growth phase and at appropriate intervals, centrifuged for 2 min at 16 000 *g* and then filtered the supernatant through a 0.2 μm membrane filter to determine the concentration of glucose, ethanol, glycerol, pyruvate, succinic acid, lactate and acetate using an HPLC 1200 Series System (Agilent Technologies). The HPLC system was fitted with a 300×7.7mm Aminex HPX−87H ion exchange Column (Biorad) thermostated at 60°C. 5 mM H_2_SO_4_ was used as a mobile phase at a flowrate of 0.6 mL/min. A refractive index detector (Agilent Technologies G1362A) set at 55°C in series with a variable wavelength detector at 210 nm (Agilent Technologies G1314B) was used for detection of compounds. Finally an Agilent ChemStation was used to compute the concentrations corresponding to calibration curves from metabolite standards (Sigma Aldrich), using a multiple point calibration system for precision and accuracy. From this data we calculated product yields, maximum specific rates of glucose consumption and production of other metabolites as reported [32]. After exhaustion of glucose in the fermenters we analyzed the disappearance of accumulated ethanol and acetate using the same procedures. The switch time from glucose to ethanol utilization was analyzed using the gas analyzer profiles and HPLC data.

### Other growth experiments

We investigated the effects of antibiotics and dead bacteria on yeast. Yeast strains were grown overnight and used to inoculate 20mL YPD (0.5% yeast extract, 1% peptone, 2% glucose, pH 6.2 supplemented with 100 μg/mL of Streptomycin) in 250-mL baffled bottom flasks and incubated as described above. To investigate the effects of dead bacteria on yeast, we heat-killed bacteria by incubating them for 5 hrs at 60°C and then co-cultured them with yeast. We also tested the growth of bacteria in acetate as a sole carbon source (0.5% yeast extract, 1% peptone, supplemented with 1, 2 or 5 g/L of acetate, pH 6.2). Cell growth was monitored by measuring OD_600nm_. All experiments were done in triplicate.

### Whole genome and RNA sequencing

Genomic DNA was extracted using methods previously described [33]. Concentrations of purified nucleotides were determined at 260 nm using a NanoDrop 2000 spectrophotometer (Thermo Scientific), and purity was assessed at absorbance ratios of 260/280 nm and 260/230 nm. DNA fragment size was determined using the Bioanalyzer 2100 (Agilent Technologies). Genomic DNA was then fragmented to an average size of 550 bp using a Covaris M220 ulltrasonicator (Covaris). Fragments were subsequently end-repaired and A-tailed, and indexed adapters were ligated. The products were purified and enriched with PCR to create the final cDNA library. The 2 tagged cDNA libraries were pooled in equal ratios and used for 2 × 100 bp paired-end sequencing on a single lane of the Illumina MiSeq (Illumina) using MiSeq Reagent kit v2 at the core sequencing facility of Academia Sinica, Taiwan.

MAC, RYT and the ancestral strains were grown in triplicates in minimal defined media supplemented with 20 g/L of glucose in batch cultures in fermenters as described above. We sampled the three batch cultures for RNA extraction between 10–11 g/L (i.e., 1%) of residual glucose under both aerobic and anaerobic conditions. In addition, we also extracted samples after the diauxic shift (approximately 0.02 g/L of residual glucose and 1.48 ± 0.02–1.74 ± 0.04 g/L of residual ethanol) to determine changes in expression of genes responsible for carbon utilization in the evolved strains. S6 Table shows residual carbon source and metabolites in the fermenters when the samples were withdrawn for RNA extraction in all three conditions. Cells were pelleted by centrifugation at 4°C for 2 min at 16 000 *g* and instantly frozen in liquid nitrogen and stored at −80°C before extraction of RNA. RNA was extracted in triplicates from cells from each of the aerobic, post-diauxic and anaerobic conditions (total 9 (triplicate for each condition) x 3 (ancestor, RYT, MAC) = 27 samples) using an RNAeasy kit (Qiagen) according to the manufacturer’s instructions. DNase treatment was performed using a Qiagen on-column kit. RNA quantity and quality was determined using a Nanodrop ND 1000 spectrophotometer (Thermo Scientific) and a Bioanalyzer 2100 (Agilent Technologies), respectively.

The cDNA libraries were constructed using the TruSeq RNA Sample Preparation Kit (Illumina) according to the manufacturer's instructions. Poly-A containing mRNA was purified from 2 μg of total RNA using oligo(dT) magnetic beads and fragmented into 200–500 bp pieces using divalent cations at 94°C for 5 min. The cleaved RNA fragments were copied into first strand cDNA using SuperScript II reverse transcriptase (Life Technologies) and random primers. After second strand cDNA synthesis, fragments were end repaired and A-tailed, and indexed adapters were ligated. The products were purified and enriched with PCR to create the final cDNA library. The 27 tagged cDNA libraries were pooled in equal ratios and used for 2 × 100 bp paired-end sequencing on a single lane of the Illumina HiSeq2000 II at the core sequencing facility of Academia Sinica, Taiwan.

### SNP and InDel detection

Raw paired-end reads from the ancestor and MAC strains were trimmed using the trim function in the CLC Genomics Workbench V7.0. The resulting FASTQ files were culled of any reads that occurred in only one read of the pair. Paired-end reads were mapped to CBS 3082 the reference genome of *L*. *kluyveri*, downloaded from (http://www.genolevures.org/download.html#sakl) with BWA-Mem function of SAMtools V0.1.19. using default parameters plus -I -q 10 and a sorted BAM file was created with SAMtools V0.1.19. Next, we marked duplicate reads with Picard version 1.44 in MarkDuplicate module in GATK V3.5. Realignment and recalibration was done using GenomeAnalysisTK module in GATK V3.5. We generated a list of candidate SNPs and indels by applying GATK’s UnifiedGenotyper V3.5. A minimum phred-scaled confidence threshold for GATK to call a mutation was set to 4.0 and the mutation to be supported by at least 6 reads.

To refine our set of candidate variants we determined the SNPs and indels from triplicate clonal RNA sequencing from all the three experimental conditions and those in whole genome sequencing. A real variant should be present in both RNA sequencing and whole genome sequencing. For RYT strains, we used RNA sequencing data from all the three experimental conditions to determine SNPs. A variant was considered as real if it was found in all the three conditions.

### Transcriptome analysis

After successfully characterizing the physiological changes that occurred when *L*. *kluyveri* was evolved with and without the presence of a bacterial selection pressure, we next examined differences between the transcriptomes of MAC and the RYT strains with respect to the ancestral strain in the three different growth conditions: aerobic, post-diauxic and anaerobic.

The sequenced reads from triplicate sequencing of the three yeasts (*i*.*e*., 27 samples) were separately annotated onto genes of the reference genome of *L*. *kluyveri* (CBS 3082) using CLC Genomics Workbench V7.0 with the following parameters: strand specific: both; similarity fraction: 0.8; length fraction: 0.8; mismatch cost: 2; insertion cost: 3; deletion cost: 3. The paired-end reads were mapped using an insert range of 72 bp—290 bp. RPKM were determined for annotated transcripts using the tools in the Transcriptomics Analysis toolbox of CLC Genomics Workbench [34]. To reduce artifacts in expression levels, only unique hits greater than 90% confidence interval (lower bound) in all the three repeats of sequencing experiment were used to determine RPKM, as a measure of expression level in this study. Significant fold change values synonymous to difference in expression levels between RYT and ancestor as well as MAC and ancestor were determined using Empirical analysis of DGE algorithm (EDGE-test) in CLC Genomics Workbench. A pair of genes in evolved yeasts was considered significantly misregulated if the absolute-value (fold change) > 1.5 and FDR corrected p-value < 0.05 (EDGE-test). The significant fold change values presented in S7 Table, has downregulated genes with fold Change < -1.5 and upregulated genes with fold Change > 1.5, for ease in interpreting the data [33–35].

### Gene function enrichment analysis

Misregulated genes in aerobic, post-diauxic and anaerobic conditions from MAC and RYT strains and were classified into upregulated and downregulated set and gene class function enrichment analysis was performed using SGD GO slim mapper [36]. Both manually curated and high-throughput data was used during this analysis. Gene sets were defined by GO terms from all three ontologies (process, function, and location). The statistical significance was assessed by performing Bonferroni correction on the p-values of the enriched functions. A gene class was considered functionally enriched if the adjusted p-value < 0.05.

### Test for directional selection analysis of gene expression

To ascertain pathways affected by evolution of *L*. *kluyveri* under MAC and RYT treatments, we designed a test, which takes into account the directional changes of gene expression in all the curated biochemical pathways available in *Saccharomyces* Genome Database [36]. The input of this analysis was the average fold change values of genes from triplicate RNASeq experiments that were significantly misregulated in MAC or RYT strains according to EDGE test. The test was performed independently on all the three conditions (Aerobic, Postdiauxic and Anaerobic).

We assigned each *L*. *kluyveri* gene one of three values based on its expression compared to the ancestor. 1 for up regulation, -1 for down regulation and 0 for no change. We analyzed the 154 curated biochemical pathways in *Saccharomyces* Genome Database, we eliminated biochemical pathways with less than 4 genes in *L*. *kluyveri* resulting in 118 pathways for the analysis. We generated scores for each pathway by summing the gene values across each pathway. Because biochemical pathways have various numbers of genes we generated null distributions for each size class of biochemical pathway (i.e., 10 genes for glycolysis, or 21 genes for the TCA cycle). Care was taken to exclude the specific pathway genes in the simulated null distribution data set. Hundred thousand random permutations were performed across the transcriptome of *L*. *kluyveri* for each pathway, using the same number of genes in each pathway. Summing the gene values in each permutation generated a null distribution of scores for each pathway. To assess if the distribution of specific pathway values was significantly different from null distribution, and also determine the directionality of selection on each pathway, we computed one sided unadjusted p-values empirically by dividing the number of times a smaller or larger real pathway score was found in the null distribution by the number of simulations. The criteria of smaller or larger real pathway score was set based on the sign of the real pathway score being positive or negative. p-values were corrected for multiple testing by multiplying the p-value with the total number of unique pathways (i.e., 118). p-values were also computed for expression scores averaged over length of the pathway to ascertain that the length of the pathway does not bias the difference between the null distribution and pathway distribution. This was further confirmed by performing correlation test (Spearman correlation coefficient) between p-values and length of the pathway. Since, a several SGD pathways correspond to amino acid and DNA biosynthesis we refined the list by excluding such pathways.

### Statistical methods

For the calculation of correlation coefficients between glucose vs ethanol and glucose vs acetate, values from t = 0 until the time that ethanol or acetate began to decrease were used. For the correlation between ethanol concentrations and bacterial cell death, values from t = 0 until the decline of ethanol production were used. All statistical analysis was performed using R Statistical Software (version 2.14.0).

### Code availability

Custom Perl scripts used for data analysis are available upon request.

## Supporting information

S1 Fig Growth profiles of bacteria and *L*. *kluyveri* in various conditions. Fold change of ethanol yield (A) and growth rates (B) from 640 to 960 generations of MAC and RYT strains with respect to the ancestor measured at every 80 generations. (C-F) Viability of other bacteria used in evolution experiment during co-culture with RYT, MAC and ancestral strains. The bactericidal activity of MAC strains was also evident on *B*. *subtilis*, *S venezuelae* and to a lower extent on *L*. *lactis*. However, no such effect was observed on *S*. *plymuthica*. (G) Specific growth rates in shake flask experimental environment. (H) Different concentrations of acetate (1, 2 or 5 g/L) were tested as a sole carbon source for *P*. *agglomerans*. 1g/L of acetate sustained growth of bacteria although with a lag phase increasing with amounts of acetate as compared to 2% glucose (control). (I) Effects of Streptomycin and ghosts bacteria on the yeast, *L*. *kluyveri*. M-R: t-test between MAC and RYT and M-A: t-test between MAC and ancestor. *: *p* < 0.09, **: *p* < 0.05, ***: *p* < 0.01. Error bars are one standard deviation.
(TIF)

S2 Fig Time course *L*. *kluyveri* growth profiles (CFUs) with and without bacterial competition. The CFU measurements of Ancestor, MAC and RYT strains show that the yeast populations are not dying along with the bacteria.
(PDF)

S3 Fig Glucose concentrations in MAC and RYT strains are anti-correlated with ethanol and acetate concentrations. (PDF)

S4 Fig Fermentation parameters in anaerobic condition. (A) Specific growth rates, (B) specific yields of ethanol, biomass, pyruvate, acetate and glycerol for ancestor (green), RYT (blue) and MAC (orange) strains in anaerobic condition. MAC strains had significantly higher growth rate and ethanol yield than both ancestor and RYT strains (t-test, *p* < 0.05). MAC strains did not produce acetate. The other metabolites’ yields in RYT and MAC strains were nearly indistinguishable from the progenitor strain. Error bars are one standard deviation.
(PDF)

S5 Fig Maximum specific rates of consumption of glucose and production of metabolites for ancestor, RYT and MAC strains. Specific rates of glucose and O_2_ consumption, and of production of acetate, glycerol, CO_2_ and ethanol and respiratory coefficients calculated during the exponential phase in aerobic (A) and anaerobic (B) conditions (ancestor (green), RYT (blue) and MAC (green)). Error bars are one standard deviation.
(PDF)

S6 Fig Time course metabolite profiles of MAC, RYT and ancestral strains in controlled aerobic condition. (PDF)

S1 Table Pathways enrichment in the direction selection analysis. Compilation of *p*-values from test for selection based on the directional changes of gene expression across biochemical pathways in both MAC and RYT strains. Glycolysis was strongly enriched along with ergosterol biosynthesis and folate biosynthesis pathways. The values presented here are associated with Fig 4A.
(XLSX)

S2 Table Overview of NGS data. Global information on whole genome sequencing and RNA sequencing data statistics.
(XLSX)

S3 Table Summary of SNP data. (A) SNPs common to WGS data from a single MAC clone and all the RNA sequencing data were analyzed. MAC strain has mutations in genes related to ethanol tolerance (highlighted in red). (B) SNPs common to RNA sequencing data from aerobic, postdiauxic and anaerobic conditions of RYT strains are presented. RYT strains have mutations in genes involved in DNA damage response, cell wall integrity and ER maintenance and oxidative stress response.
(XLSX)

S4 Table Results of GO analysis of misregulated genes in all the three conditions. (XLSX)

S5 Table Ethanol tolerance in bacteria and yeast. Bacteria were grown in LB liquid media supplemented with ethanol of different concentrations and effects of ethanol were determined by CFUs/mL counts. *L*. *kluyveri* was grown on solid YPD and ethanol effects were determined by scoring growth.
(XLSX)

S6 Table Metabolite values at RNA extraction points. (XLSX)

S7 Table Expression fold change information of all the *L*. *kluyveri* genes with *S*. *cerevisiae* orthologs. (XLSX)
