# Supplementary figures and images for: Coevolution with bacteria drives the evolution of aerobic fermentation in Lachancea kluyveri
Source: PLoS One. 2017 Mar 10;12(3):e0173318. doi: 10.1371/journal.pone.0173318 (PMC5345805; doi:10.1371/journal.pone.0173318)

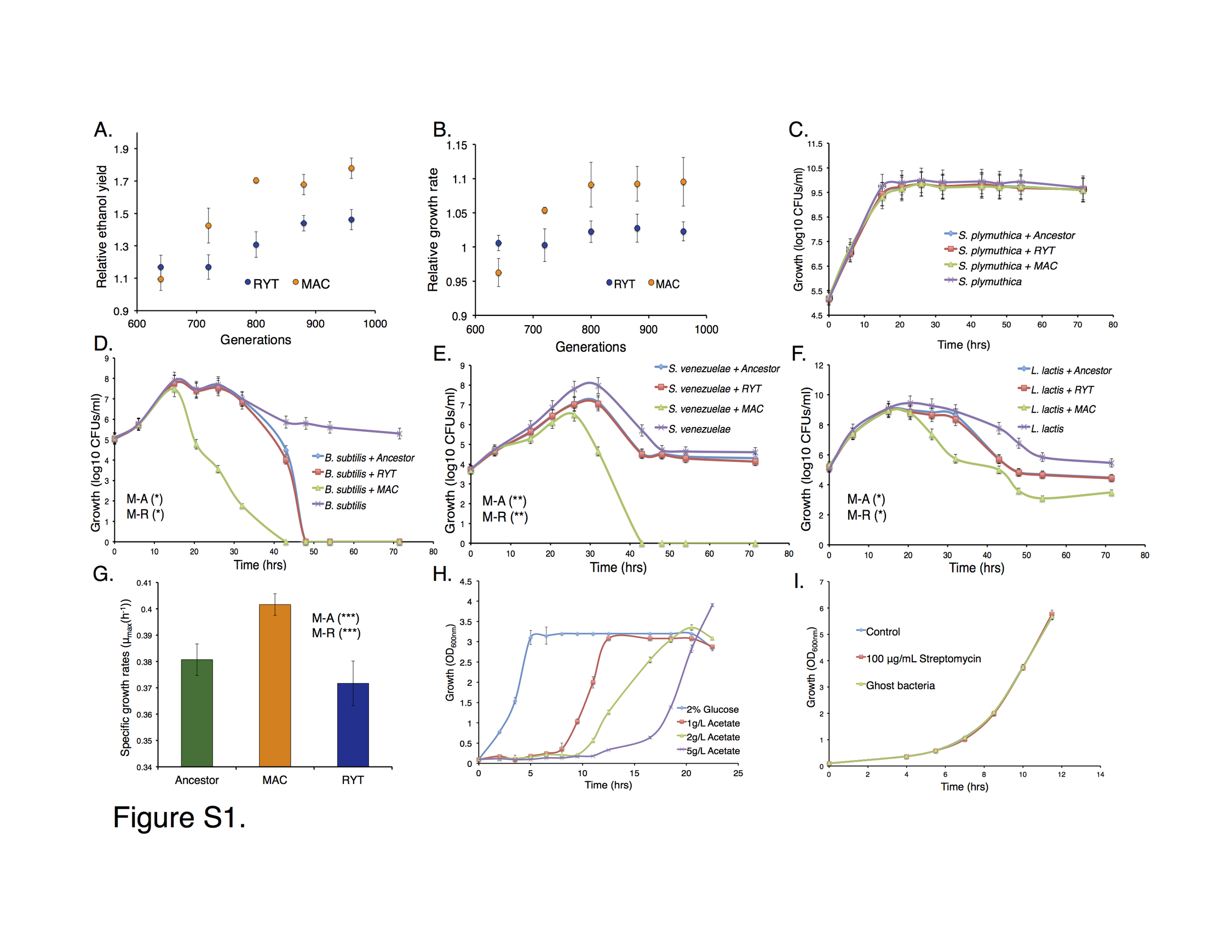

Supplement: S1 Fig — Fold change of ethanol yield (A) and growth rates (B) from 640 to 960 generations of MAC and RYT strains with respect to the ancestor measured at every 80 generations. (C-F) Viability of other bacteria used in evolution experiment during co-culture with RYT, MAC and ancestral strains. The bactericidal activity of MAC strains was also evident on B. subtilis, S venezuelae and to a lower extent on L. lactis. However, no such effect was observed on S. plymuthica. (G) Specific growth rates in shake flask experimental environment. (H) Different concentrations of acetate (1, 2 or 5 g/L) were tested as a sole carbon source for P. agglomerans. 1g/L of acetate sustained growth of bacteria although with a lag phase increasing with amounts of acetate as compared to 2% glucose (control). (I) Effects of Streptomycin and ghosts bacteria on the yeast, L. kluyveri. M-R: t-test between MAC and RYT and M-A: t-test between MAC and ancestor. *: p < 0.09, **: p < 0.05, ***: p < 0.01. Error bars are one standard deviation. (TIF) [file pone.0173318.s001.tif]

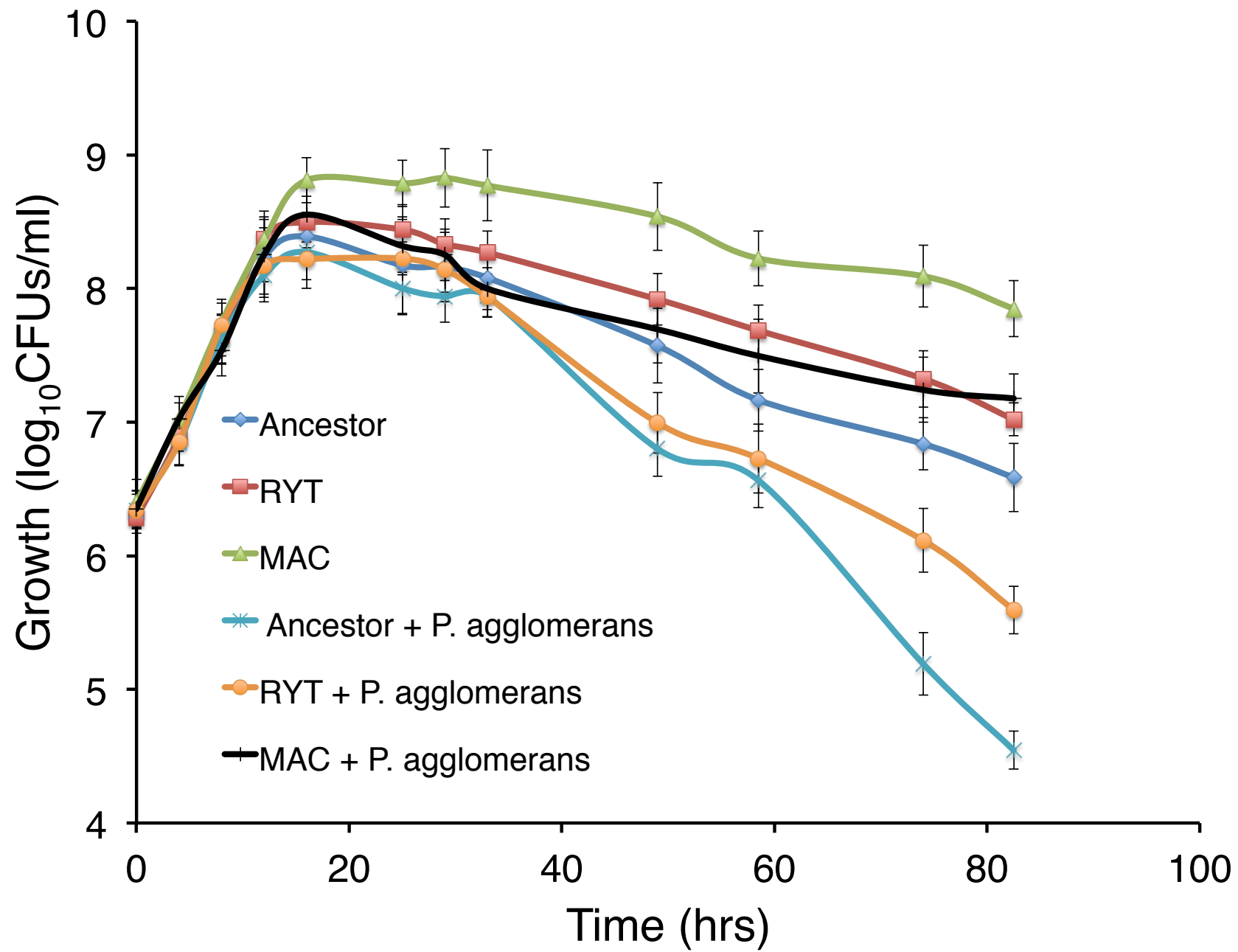

Figure S2.

Supplement: S2 Fig — The CFU measurements of Ancestor, MAC and RYT strains show that the yeast populations are not dying along with the bacteria. (PDF) [file pone.0173318.s002.pdf]

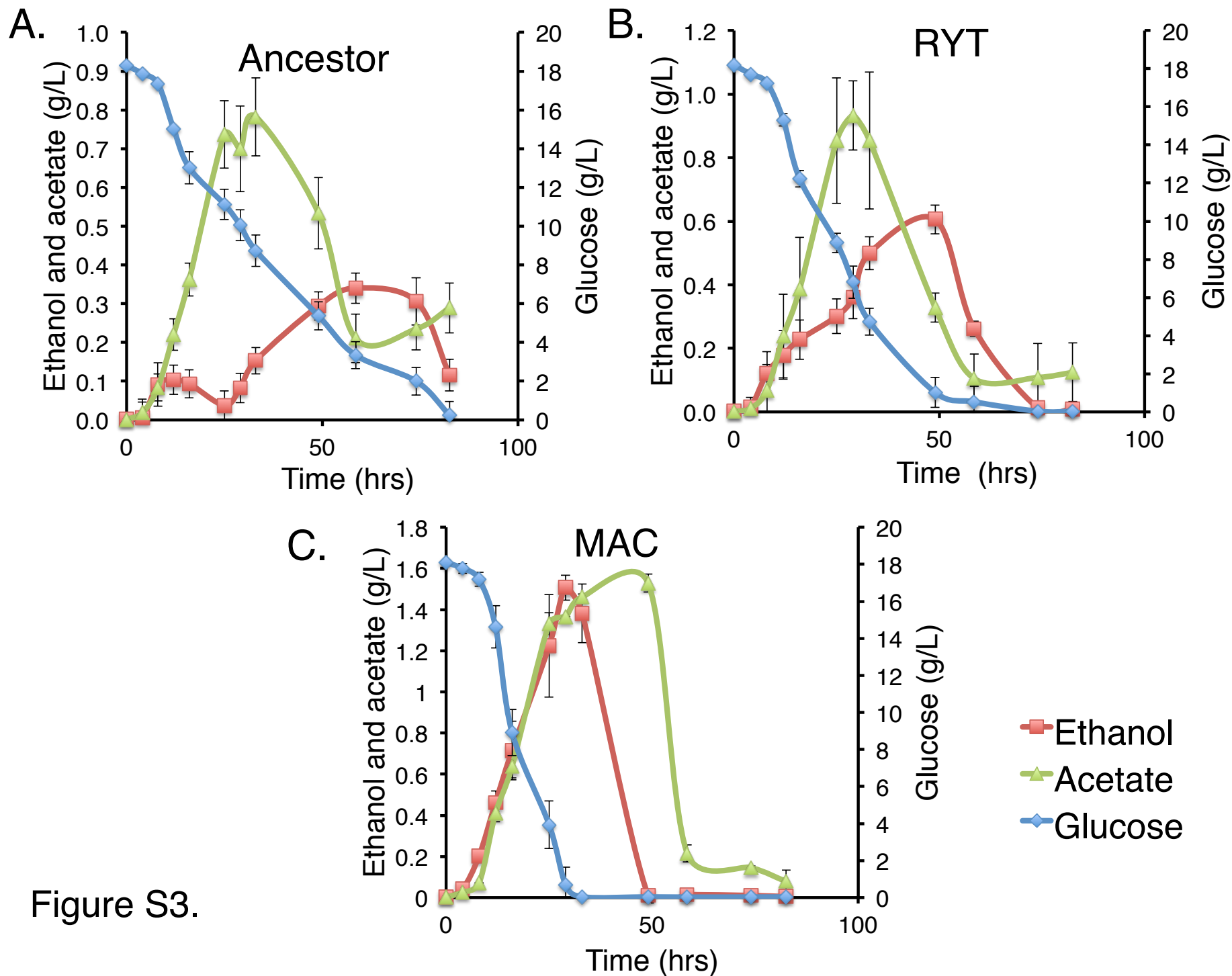

Figure S3.

Supplement: S3 Fig — (PDF) [file pone.0173318.s003.pdf]

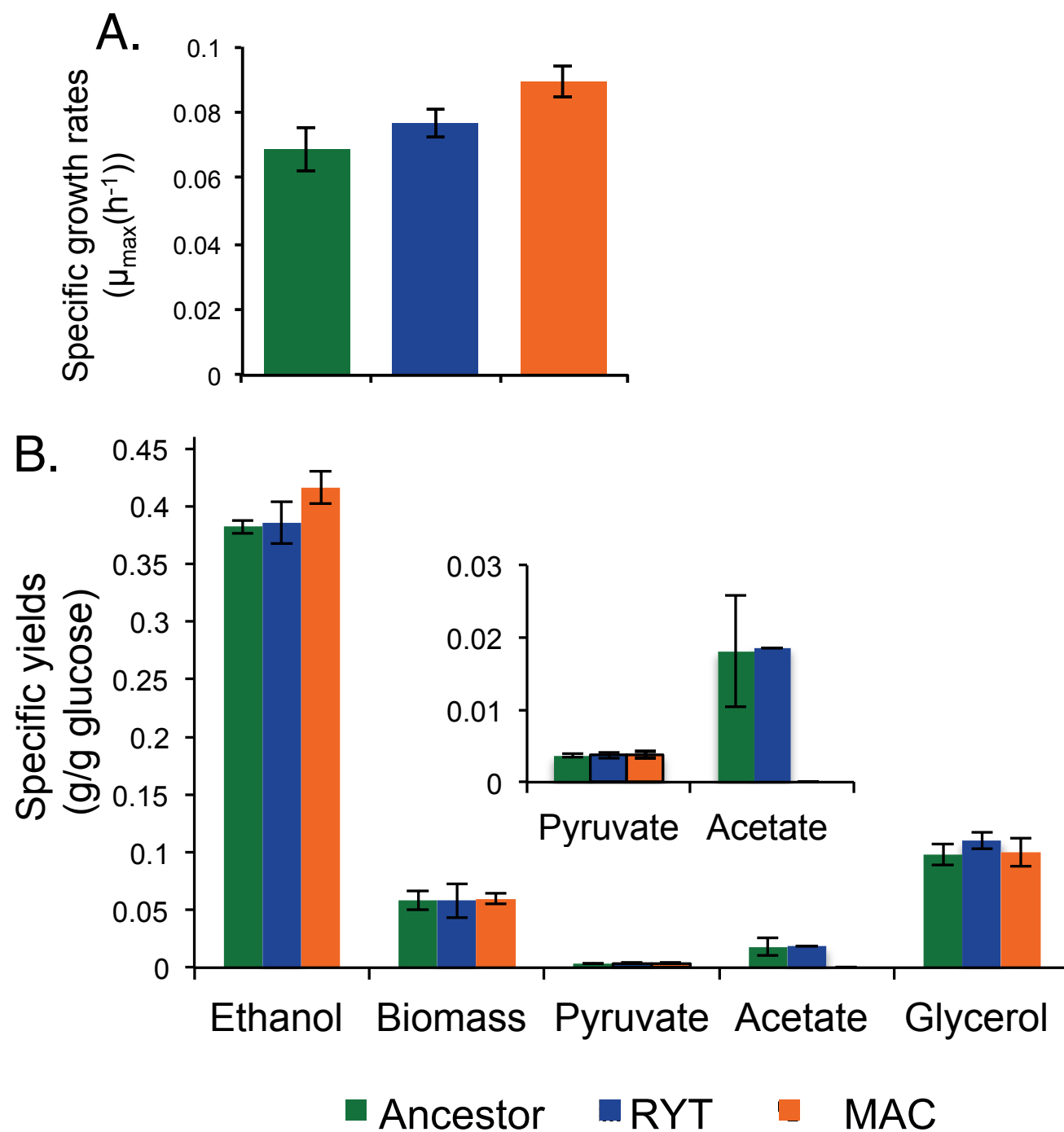

Figure S4.

Supplement: S4 Fig — (A) Specific growth rates, (B) specific yields of ethanol, biomass, pyruvate, acetate and glycerol for ancestor (green), RYT (blue) and MAC (orange) strains in anaerobic condition. MAC strains had significantly higher growth rate and ethanol yield than both ancestor and RYT strains (t-test, p < 0.05). MAC strains did not produce acetate. The other metabolites’ yields in RYT and MAC strains were nearly indistinguishable from the progenitor strain. Error bars are one standard deviation. (PDF) [file pone.0173318.s004.pdf]

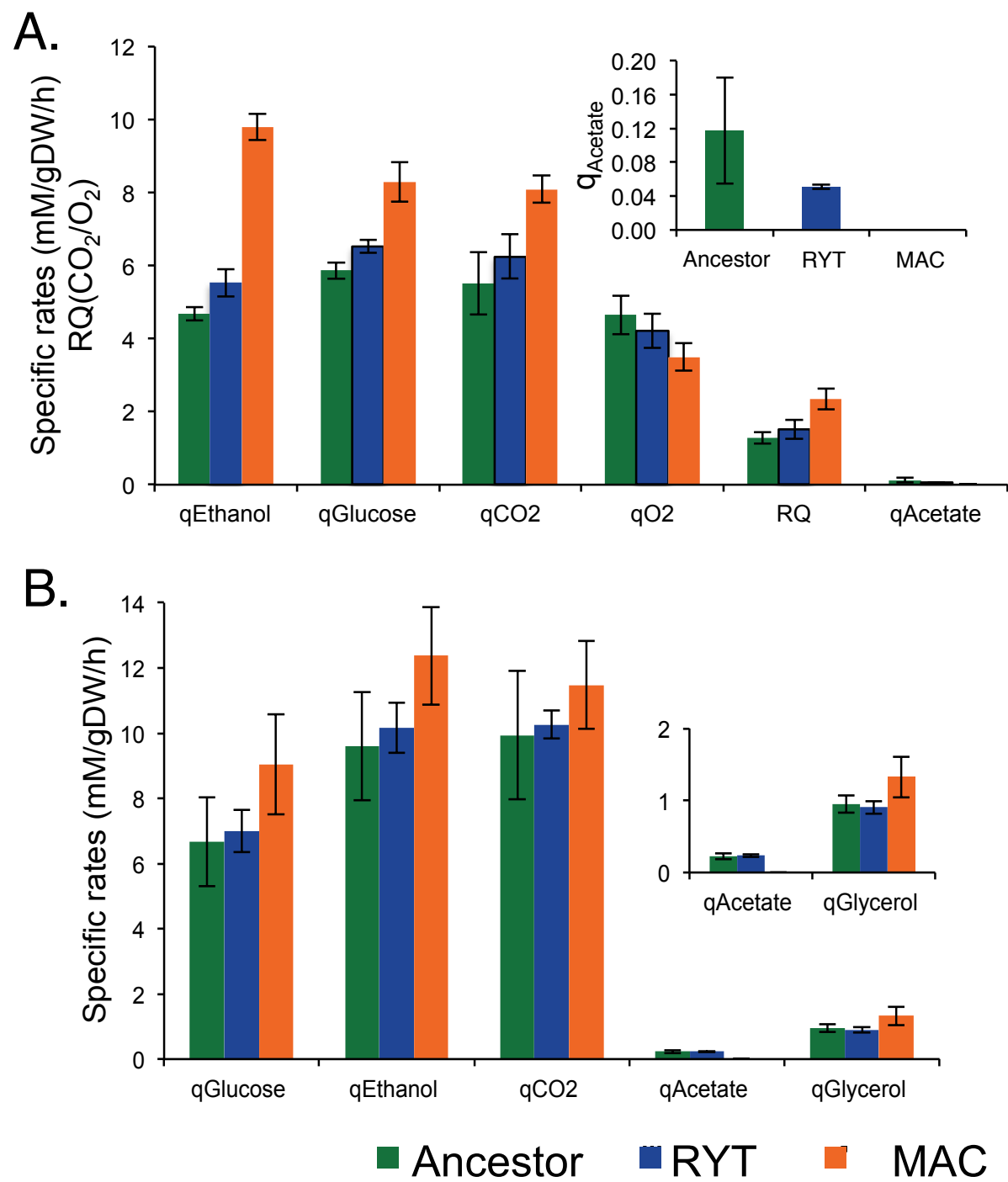

Figure S5.

Supplement: S5 Fig — Specific rates of glucose and O2 consumption, and of production of acetate, glycerol, CO2 and ethanol and respiratory coefficients calculated during the exponential phase in aerobic (A) and anaerobic (B) conditions (ancestor (green), RYT (blue) and MAC (green)). Error bars are one standard deviation. (PDF) [file pone.0173318.s005.pdf]

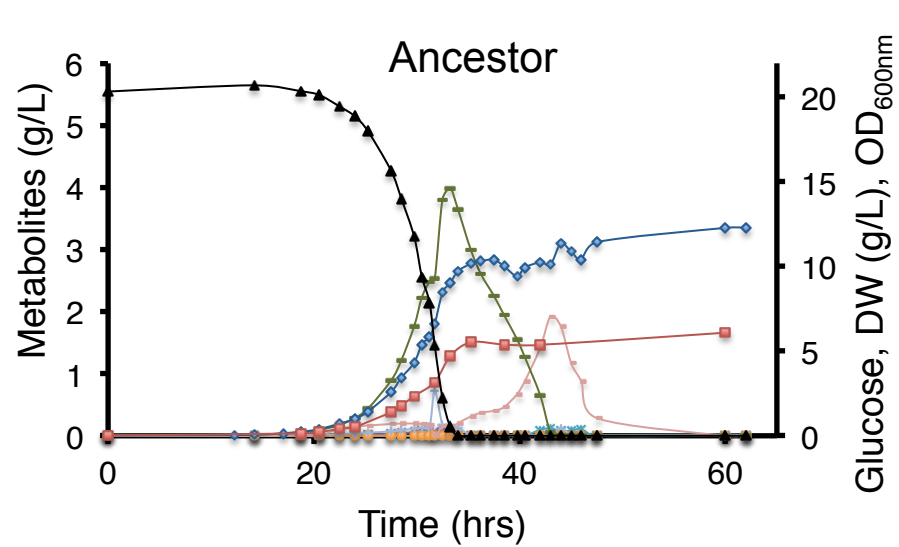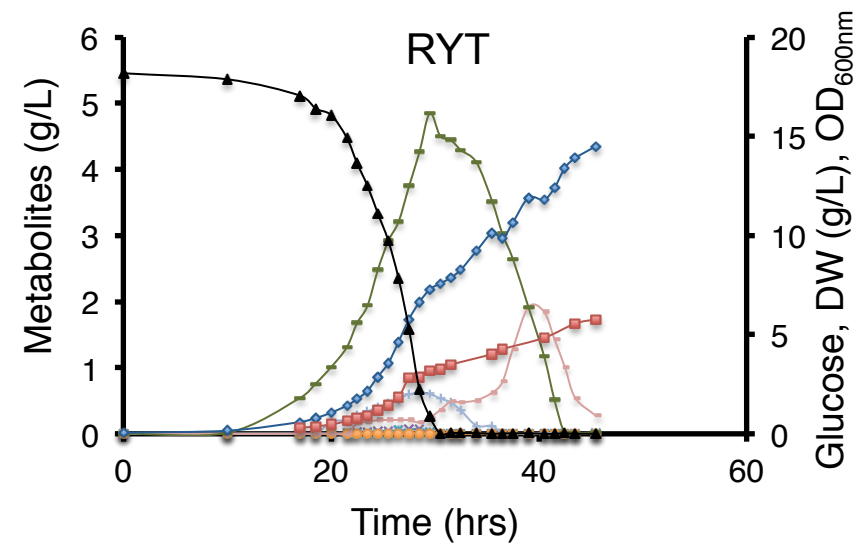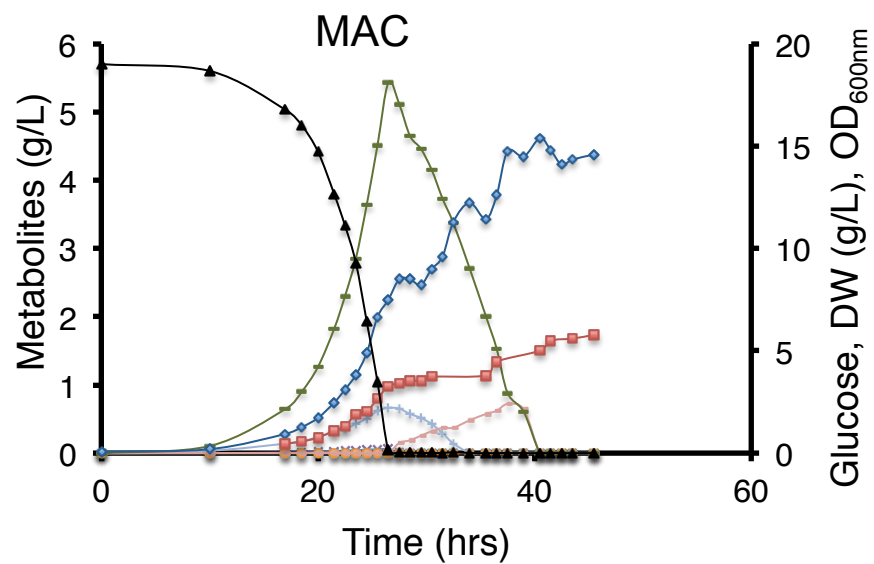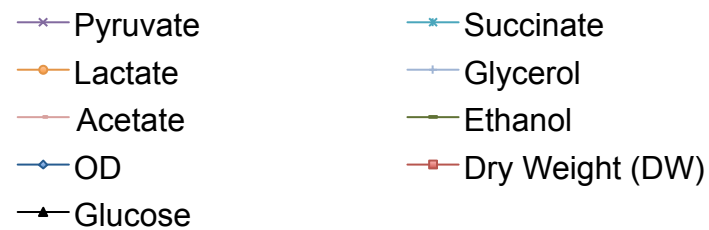

Figure S6.

Supplement: S6 Fig — (PDF) [file pone.0173318.s006.pdf]
